# “Endothelium-Out” and “Endothelium-In” Descemet Membrane Endothelial Keratoplasty (DMEK) Graft Insertion Techniques: A Systematic Review With Meta-Analysis

**DOI:** 10.3389/fmed.2022.868533

**Published:** 2022-06-14

**Authors:** Hon Shing Ong, Hla M. Htoon, Marcus Ang, Jodhbir S. Mehta

**Affiliations:** ^1^Department of Corneal & External Eye Diseases, Singapore National Eye Centre, Singapore, Singapore; ^2^Singapore Eye Research Institute, Singapore, Singapore; ^3^Duke-NUS Medical School, Singapore, Singapore; ^4^School of Materials Science and Engineering, Nanyang Technological University, Singapore, Singapore

**Keywords:** endothelial keratoplasty, Descemet’s membrane endothelial keratoplasty, DMEK, bullous keratopathy, cornea, corneal transplants, outcomes, surgical techniques

## Abstract

**Background:**

We evaluated the visual outcomes and complications of “endothelium-out” and “endothelium-in” Descemet membrane endothelial keratoplasty (DMEK) graft insertion techniques.

**Materials and Methods:**

Electronic searches were conducted in CENTRAL, Cochrane databases, PubMed, EMBASE, ClinicalTrials.gov. Study designs included clinical trials, comparative observational studies, and large case series (≥25 eyes). PRISMA guidelines were used for abstracting data and synthesis. Random-effects models were employed for meta-analyses.

**Results:**

21,323 eyes (95 studies) were included. Eighty-six studies reported on “endothelium-out” techniques; eight studies reported on “endothelium-in” techniques. One study compared “endothelium-out” to “endothelium-in” techniques. Eighteen “endothelium-out” studies reported that 42.5–85% of eyes achieved best-corrected visual acuity (BCVA) ≥20/25 at 6 months; pooled proportion of eyes achieving BCVA ≥20/25 at 6 months was 58.7% (95% CI 49.4–67.7%,15 studies). Three “endothelium-in” studies reported that 44.7–87.5% of eyes achieved BCVA of ≥20/25 at 6 months; pooled proportion of eyes achieving BCVA ≥20/25 at 6 months was 62.4% (95% CI 33.9–86.9%). Pooled mean endothelial cell loss was lower in the *“endothelium-in”* studies (28.1 ± 1.3%, 7 studies) compared to *“endothelium-out”* studies (36.3 ± 6.9%,10 studies) at 6 months (*p* = 0.018). Graft re-bubbling rates were higher in the “endothelium-out” studies (26.2%, 95% CI 21.9–30.9%, 74 studies) compared to “endothelium-in” studies (16.5%, 95% CI 8.5–26.4%, 6 studies), although statistical significance was not reached (*p* = 0.440). Primary graft failure rates were comparable between the two groups (*p* = 0.552). Quality of evidence was considered low and significant heterogeneity existed amongst the studies.

**Conclusion:**

Reported rates of endothelial cell loss were lower in “endothelium-in” DMEK studies at 6 months compared to “endothelium-out” studies. Outcomes of “endothelium-in” techniques were otherwise comparable to those reported in “endothelium-out” studies. Given the technical challenges encountered in “endothelium-out” procedures, surgeons may consider “endothelium-in” techniques designed for easier intra-operative DMEK graft unfolding. “Endothelium-in” studies evaluating outcomes at longer time points are required before conclusive comparisons between the two techniques can be drawn.

## Introduction

### Background

Loss of vision from diseases of the corneal endothelium is the predominant indication for corneal transplantations (1, 2). Over the past 20 years, selective replacement of damaged corneal endothelium using lamellar keratoplasty procedures has significantly changed the management of endothelial diseases (3–5). The first posterior lamellar keratoplasty procedure was described in the late 1990s (6). In this report, the surgeon only partially replaced the recipient’s diseased corneal endothelium, avoiding full-thickness or penetrating keratoplasty (PK). Ensuing developments to the procedure have resulted in more advanced techniques of endothelial keratoplasty (EK), which are associated with better visual outcomes, lower graft rejection risks, and improved graft survival rates (5, 7–9). Unlike PK, these EK techniques avoid full-thickness corneal trephination and intra-operative “open sky” situations associated the risks of severe blinding complications such as suprachoroidal hemorrhage. Endothelial keratoplasties also maintain corneal biomechanics and the overall strength of the globe, important in protecting the eye from external trauma. Data from national corneal graft registries have reported that EK procedures have now overtaken full-thickness PK as the leading procedure for treating corneal endothelial diseases in several countries (1, 2, 10).

Currently, there are two predominant techniques of EK performed worldwide: Descemet’s stripping automated endothelial keratoplasty (DSAEK) and Descemet membrane endothelial keratoplasty (DMEK) (3, 4, 11). In DSAEK, the transplanted corneal grafts are comprised of donor endothelium, Descemet’s membrane (DM), and some posterior stroma. Advancement of the DSAEK technique, such as the development of devices for graft insertion and techniques to cut thinner grafts, has greatly simplified DSAEK (12–15). With more predictable visual outcomes and faster visual recovery compared to PK (8, 16, 17), many corneal surgeons are now performing DSAEK as the primary technique to treat end-stage corneal endothelial diseases (18, 19).

Descemet membrane endothelial keratoplasty is the more recent advancement in EK surgery (20). In DMEK, only the DM and the corneal endothelium are harvested from donor corneal tissues and transplanted, rendering them anatomically more accurate. As corneal stroma is not transplanted, changes in corneal profiles are avoided. Faster visual recovery and improved visual outcomes compared to DSAEK can thus be achieved (21–25). Lower rates of graft rejection have also been reported in DMEK compared to DSAEK (26).

### Rationale for This Review

Current methods of DMEK graft transfer into the anterior chamber involve inserting the graft through a small clear corneal wound. Different surgical instruments have been described for the insertion of DMEK grafts. Examples of such instruments include glass injectors (27, 28) and intraocular lens cartridges (29, 30). All these instruments are designed to shield the DMEK graft scroll from the surgical wound. Nevertheless, the majority of techniques reported in published literature involves the loading and insertion of the DMEK graft with the endothelium on the outer surface (“endothelium-out”). Thus, the grafts are potentially at risk of endothelial cell loss due to endothelial contact with the walls of the injection devices. Furthermore, “endothelium-out” DMEK techniques all involve the injection of the entire scrolled graft into the anterior chamber. The un-scrolling of the free floating graft, following its insertion, can be difficult and unpredictable (31, 32). Such challenges have hindered corneal surgeons from adopting DMEK as a primary treatment for corneal endothelial failure (2, 3). In a recent eye banking report, DSAEK still accounted for over 55% of EK procedures performed in the United States (2).

**FIGURE 1:**
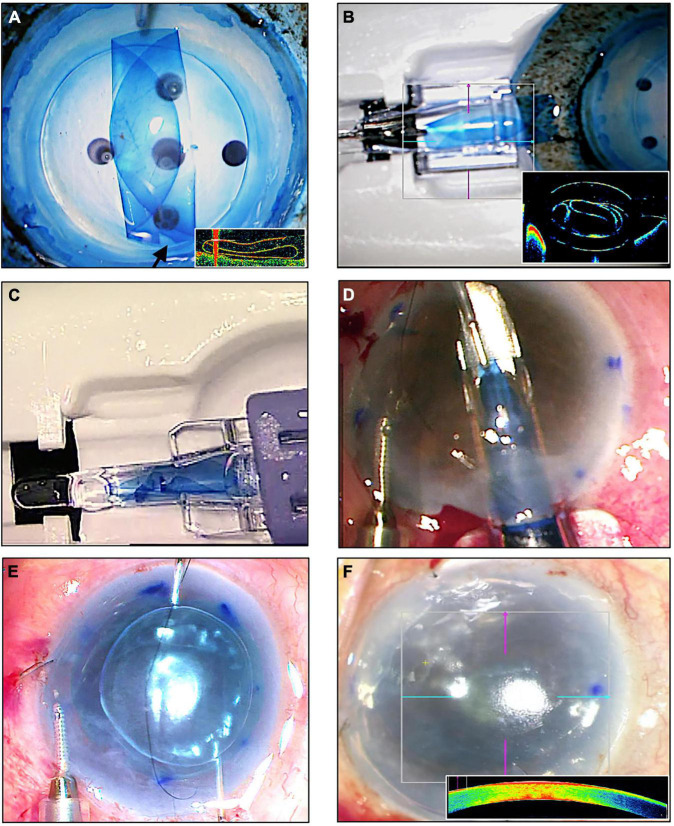
An “endothelium-in” surgical technique of Descemet membrane endothelial keratoplasty (DMEK) using the using the DMEK EndoGlide (Network Medical Products, United Kingdom). **(A)** DMEK graft is folded into a tri-fold with its endothelium in its inner surface; note the asymmetrical orientation marker (arrow); (inset) intraoperative optical coherence tomography (OCT) image of the tri-folded graft – note that the leaves of the tri-fold do not touch. **(B)** Graft is pulled and loaded into the EndoGlide; (inset) OCT image showing the tri-folded graft within the DMEK EndoGlide – note that the leaves of the tri-fold do not touch. **(C)** Customized clip fixed to the back of the EndoGlide; this creates a “closed system” after graft insertion maintaining anterior chamber stability. **(D)** Graft is drawn into the anterior chamber with micro-forceps with its endothelium facing down. **(E)** Unfolding of the graft with its orientation maintained whilst air is injected for tamponade. **(F)** Full air-gas tamponade of graft; (inset) intraoperative OCT showing a fully attached DMEK graft.

“Endothelium-in” DMEK graft insertion techniques have been described more recently (33–37) (Figure 1). In these techniques, the harvested DM is folded and prevented from adopting its natural scroll with its endothelium on the outside. By maintaining the orientation of the DMEK graft during graft insertion, these “endothelium-in” techniques aim to provide more control in graft unscrolling following insertion into the eye. Nevertheless, the differences in surgical outcomes of either technique for DMEK graft insertion, “endothelium-in” or “endothelium-out,” remains unclear.

### Objectives of This Review

This review aims to evaluate the published literature reporting the visual outcomes and complications of both “endothelium-out” and “endothelium-in” graft insertion techniques for DMEK.

## Materials and Methods

This review was submitted to PROSPERO International prospective register of systematic reviews (reference ID: 160657)^1^. A study protocol for this systematic review is available in Supplementary Appendix 1.

### Criteria for Considering Studies for This Review

#### Types of Intervention

We included publications in which the visual outcomes and complications of DMEK performed for the treatment of endothelial dysfunction were reported.

#### Types of Studies

Study designs included controlled clinical trials, prospective or retrospective comparative observational studies, and large case series (≥25 eyes). Small case series (<25 eyes), letters, reviews, published abstracts, and laboratory-based studies were excluded.

#### Types of Participants (Study Population)

Studies reporting only surgical outcomes of DMEK performed for graft failure (including repeat DMEK surgery) or specific high-risk disease groups (e.g., glaucoma, cytomegalovirus endotheliitis, herpes simplex) were also excluded. To avoid duplicate reporting of similar study populations, where the same group of investigators published several studies, earlier smaller studies were excluded if more recent larger studies reporting the same outcome measures were available.

### Information Sources

Information sources included all applicable electronic databases, all relevant articles in the reference list of any relevant articles, and all relevant articles which cite any relevant articles.

### Search Methods for Identification of Studies

Electronic literature searches were conducted in the following databases: CENTRAL, Cochrane Library databases^2^, PubMed, EMBASE, ClinicalTrials.gov.^3^ No date or language restrictions were set in our electronic searches. Key search terms were the MeSH headings Descemet’s membrane endothelial keratoplasty, Descemet membrane endothelial keratoplasty, and DMEK. The last electronic database search was performed on 30 June 2021. The search strategies for the relevant databases can be found in Supplementary Appendix 2.

### Data Collection and Analysis

#### Selection of Studies

Citations and abstracts obtained from electronic searches were examined. Replicated studies and evidently irrelevant studies were removed. Full text prints of relevant studies were retrieved; they were assessed against our inclusion criteria for this review.

#### Data Extraction and Management

Only data from eyes that had received DMEK surgeries were included. Where studies reported on the outcomes of eyes that had undergone surgeries other than DMEK, these eyes were excluded from the review. The following details of each study were extracted for this review: study participants’ characteristics, study design, DMEK graft insertion techniques, and surgical outcome measures.

#### Assessment of Risks of Bias in Included Studies

The study design of each article was assessed and rated according to its level of evidence. A rating scale adapted from the Oxford Centre for Evidence-Based Medicine was used (38) (Table 1).

**TABLE 1:** Level of evidence used to rate the design of each study (adapted from the Oxford Centre for Evidence-Based Medicine March) (38).

Level of evidence	Study design
1	Well-designed and conducted RCT
2	Cohort studies and low quality RCT (e.g., <80% follow-up)
3	Case-control studies
4	Case-series and poor quality^†^ cohort studies or case-control studies

*RCT, randomized controlled trials.*

*^†^Poor quality cohort study indicate one that failed to clearly define comparison groups and/or failed to measure exposures and outcomes in the same (preferably blinded), objective way in both exposed and non-exposed individuals and/or failed to identify or appropriately control known confounders and/or failed to carry out a sufficiently long and complete follow-up of patients; poor quality case-control study indicate one that failed to clearly define comparison groups and/or failed to measure exposures and outcomes in the same (preferably blinded), objective way in both cases and controls and/or failed to identify or appropriately control known confounders.*

Studies meeting the inclusion criteria were also assessed for risk of bias using Chapter 8 of the *Cochrane Handbook for Systematic Reviews of Intervention* (39). The following domains for potential risk of bias were considered: (a) *selection bias* – random sequence generation, (b) *selection bias* – allocation concealment, (c) *performance/detection bias* – masking of outcome examiners and participants (to determine whether knowledge of the allocated intervention was adequately prevented during the study), (d) *attrition bias* incomplete outcome data, and (e) *reporting bias* – selective outcome reporting. Each study was graded as “low risk” of bias, “high risk” of bias, or “unclear risk.” Any differences between the authors were resolved by discussion.

#### Outcome Measures

Data on the following surgical outcome measures were obtained: visual outcomes, endothelial cell loss, and complications including graft detachment/re-bubbling, graft rejection, and graft failure. For direct comparison of visual outcomes, measures of visual acuities in Snellen were converted to the respective logarithm of the minimum angle of resolution (LogMAR) equivalents. The proportion of eyes that achieved a best-corrected visual acuity (BCVA) of 20/25 or better at a specific time points were also evaluated.

#### Measures of Treatment Effect

All outcome measures (proportion of eyes achieving ≥20/25 BCVA, re-bubbling rates for graft detachments, graft rejection rates, and graft failure rates) were discrete data, except mean endothelial cell loss where outcome measures were continuous data. Outcomes for eyes rather than individuals were used as the unit of analysis. Studies where both eyes received the same treatment were included.

#### Managing Missing Data

All relevant data were extracted from the published studies. These included the details of studies and their quantitative results, without having to request these data from the original investigators.

#### Data Synthesis

Data analyses were performed according to Chapter 9 of the Cochrane Handbook for Systematic Reviews of Interventions (39). As published studies were performed in different institutions at various times, it is likely that variations exist amongst the patient populations included in this review. We therefore employed a random-effects model for our meta-analyses as the true effect size might differ between studies. Where we could not perform a meta-analysis, narrative syntheses describing the directions, magnitude, and consistencies of effects across the studies has been presented. MedCalc software was used for providing the meta-analyses results (MedCalc^®^ Statistical Software version 20.014; MedCalc Software Ltd., Ostend, Belgium; 2021).^4^

#### Assessment of Heterogeneity

We identified dissimilarities between published studies which are expected to introduce heterogeneities. As some degree of heterogeneity would always exist due to the diversities in methodologies of studies, where appropriate, we employed the Chi^2^ test and I^2^ statistic to quantify heterogeneities across reports. Significant heterogeneity was defined as an I^2^ statistic of ≥50% and a Chi^2^ test *p*-value of <0.1. If all the effects of an outcome measure were in a similar direction, then we considered data-pooling to be acceptable even in the existence of heterogeneities.

## Results

### Results of Search

**FIGURE 2:**
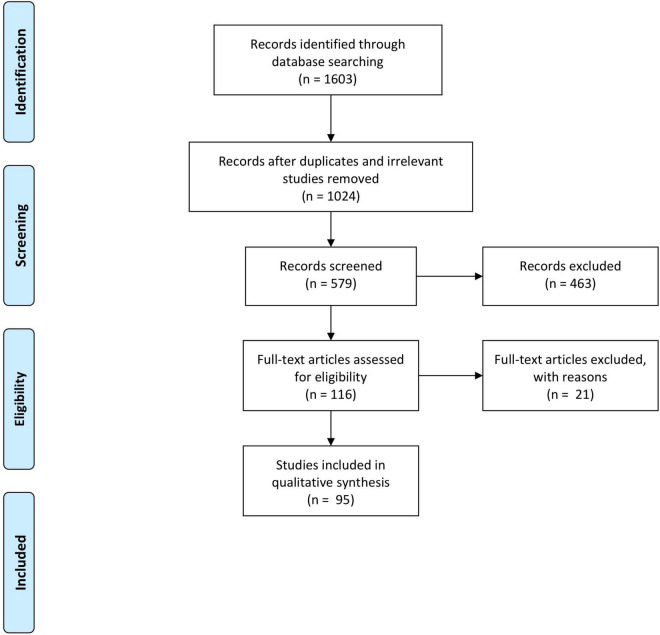
The PRISMA flow diagram.

Electronic searches generated a total of 1,603 citations. Publications not relevant to the review were removed. After removal of duplicated publications, abstracts of 579 records were screened and a further 463 records were removed. Full text copies of 116 articles were obtained and reviewed. We included 95 studies in this review; 21 studies that failed to meet the inclusion criteria were excluded. The PRISMA flow diagram is illustrated in Figure 2.

### Characteristics of Included Studies

Studies included in this review are summarized in Supplementary Table 1. A total of 21,323 eyes in 95 studies that had undergone DMEK were included. Eighty-six studies (19,945 eyes) reported on “endothelium-out” insertion techniques; eight studies (624 eyes) reported on “endothelium-in” insertion techniques, respectively. Only one study (36) compared “endothelium-out” to “endothelium-in” DMEK graft insertion techniques; this study was a large comparative series of 754 eyes (36).

### Levels of Evidence and the Risks of Bias in Included Studies

Using the Oxford Centre for Evidence-Based Medicine rating (38) of the “endothelium-out” studies included, 4/86 (4.7%) were rated level I, 17/86 (19.8%) were rated level II, 22/86 (25.6%) were rated level III, and 43/86 (50.0%) were rated level IV evidence. Of the eight “endothelium-in” studies included, 5/8 (62.5%) were rated level III evidence and 3/8 (37.5%) were rated level IV evidence. The study that included both “endothelium-out” and “endothelium-in” techniques was rated level III evidence.

**FIGURE 3:**
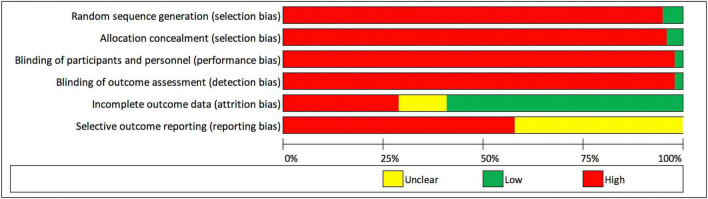
Risk of bias graph: review authors’ judgments about each risk of bias domain presented as percentages across all included studies.

Figure 3 summarizes the judgments of each risk of bias domain of all studies included. Five of 95 included studies (5.3%) were assessed as “low risk” and 90/95 (94.7%) as “high risk” of random sequence generation (selection bias). Four of 95 studies (4.2%) were assessed as “low risk” and 91/95 (95.8%) as “high risk” of allocation concealment (selection bias). Two of 95 studies (2.1%) and two studies (2.1%) were assessed as “low risk” of performance bias and detection bias, respectively. Fifty-six of 95 studies (58.9%), 28/95 (29.5%), and 11/76 (11.6%) were assessed as “low risk,” “high risk,” and “unclear risk” of attrition bias, respectively. When assessing selective reporting (reporting bias), it was noted that all included studies reported results on some of the pre-specified outcome measures for this review. No study reported results for every outcome measure. All included studies did not state whether the published methods used in the analysis of outcomes were pre-specified in a protocol. Thus, 55/95 (57.9%) and 40/95 (42.1%) of studies were assessed as “high risk” or “unclear risk” for selective reporting, respectively. The authors’ judgments of each risk of bias item for each included study is found in Supplementary Appendix 3.

### Visual Outcomes and Complications Reported in Studies

The visual outcomes and complications reported in studies included are summarized in Supplementary Table 1.

#### Follow-Up

The reported mean length of follow-up of all studies ranged from 0 to 60 months (mean 12.8 ± 12.2 months).

#### Visual Outcomes

*“Endothelium-out” studies:* Thirty-four of the 87 studies (39.1%) reported the mean BCVA at 6 months after DMEK surgery; BCVA ranged from 0.0 to 0.49 LogMAR.

Fifteen studies (17.2%) reported that 42.5–85% of eyes achieved a BCVA of 20/25 or better at 6 months. The random pooled proportion of eyes achieving BCVA of 20/25 or better at 6 months was 58.7% (95% CI 49.4–67.7%) (15 studies).

*“Endothelium-in” studies:* Two of the nine studies (22.2%) reported the mean BCVA at 6 months after DMEK surgery; BCVA ranged from 0.09 to 0.10 LogMAR. Three studies (33.3%) reported that 44.7–87.5% of eyes achieved a BCVA of 20/25 or better at 6 months. The random pooled proportion of eyes achieving BCVA of 20/25 or better at 6 months was 62.4% (95% CI 33.9–86.9%) (3 studies).

#### Endothelial Cell Loss

*“Endothelium-out” studies:* 67/87 (77.0%) studies reported data on percentage endothelial cell loss following DMEK surgery at various time points. The mean endothelial cell loss ranged from 19 to 53%. One study (40), reported a rate of 5.6–6.4% endothelial cell loss per year. The random pooled mean endothelial cell loss was 36.3 ± 6.4% at 6 months (27 studies) and 38.7 ± 7.2% at 12 months (12 studies).

*“Endothelium-in” studies:* Percentage endothelial cells loss data following DMEK surgery were reported in eight out of the nine studies (88.9%) at various time points. The reported mean endothelial cell loss range from 26.6 to 56.0%. The random pooled mean endothelial cell loss was 28.1 ± 1.3% at 6 months (7 studies) and 29.6 ± 1.2% at 12 months (1 studies).

Comparing outcomes of *“endothelium-out”* to *“endothelium-in”* techniques, pooled mean endothelial cell loss was lower in the *“endothelium-in”* group, compared to *“endothelium-out”* group at 6 months (*p* = 0.018). However, this was not statistically computable at 12 months as there was only 1 study for the “*endothelium in”* group.

#### Rates of Complications

*“Endothelium-out” studies:* Re-bubbling rates to treat DMEK graft detachments were reported in 77/87 (88.5%) studies and ranged from 0 to 82%. Fifty-eight (66.7%) studies reported primary graft failure rates which ranged from 0 to 21.0%. Thirty-five (40.2%) studies reported secondary graft failure rates which ranged from 0 to 7.0% at 15.3 ± 13.9 months. Fifty (57.5%) studies reported graft rejection rates; rates ranged from 0 to 7.0%.

*“Endothelium-in” studies:* Re-bubbling rates to treat DMEK graft detachments were reported in all nine studies and ranged from 4.7 to 45.7%. Six of the nine studies (66.7%) reported primary graft failure rates which ranged from 0 to 3.0%. Three of the nine studies (33.3%) reported on secondary graft failures rates which ranged from 0 to 6.5%.

The random pooled graft re-bubbling rates for *“endothelium-out”* and *“endothelium-in”* techniques were 26.2% (95% CI 21.9–30.9%) (74 studies) and 16.5% (95% CI 8.5–26.4%) (6 studies), respectively. Comparing outcomes of *“endothelium-out”* to *“endothelium-in”* techniques, graft re-bubbling rates were not statistically significant in the “endothelium-out” group (*p* = 0.440). The random pooled primary graft failure rates for *“endothelium-out”* and *“endothelium-in”* techniques were 2.9% (95% CI 2.03–4.02%) (58 studies) and 1.5% (95% CI 0.6–2.7%) (5 studies), respectively. Comparing outcomes of *“endothelium-out”* to *“endothelium-in”* techniques, there was no significant difference in primary graft failure rates between the two groups (*p* = 0.552).

## Discussion

**FIGURE 4:**
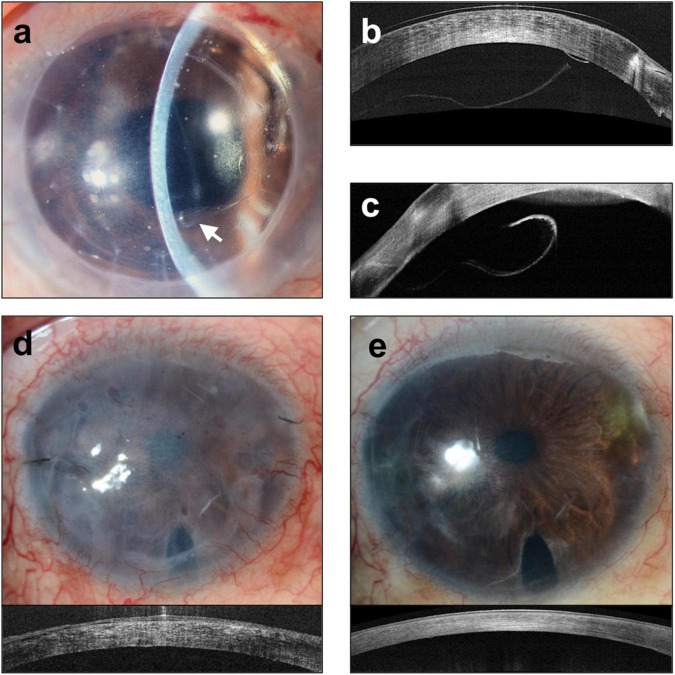
Complications of Descemet membrane endothelial keratoplasty (DMEK). **(a)** Slit lamp image of graft detachment (arrow) at post-operative day 7 and corresponding anterior segment optical coherence tomography (ASOCT) (Optovue, Oculus, CA, United States). Images **(b,c)** showing detached graft. **(d)** Iatrogenic graft failure likely a result of inadvertent graft eversion showing a hazy and thick cornea. **(e)** Repeated DMEK surgery with correct graft orientation showing rapid clearance of cornea and reduction in corneal thickness.

Although DMEK offers the advantages of faster visual rehabilitation, better visual and refractive outcomes (21–25), and lower risks of graft rejection compared to DSAEK (26), many transplant surgeons have been slow to adopt DMEK as procedure of choice for the management of endothelial diseases (2, 3). Indeed, DSAEK still accounts for approximately 57% of EK surgeries performed in the United States (2). This has been ascribed to: the technical difficulties in DMEK donor preparation and surgical technique, with the reported higher risks of early complications, namely graft detachment and iatrogenic graft failure due to inadvertent up-side-down graft (25, 26, 31, 41–45) (Figure 4). The insertion and un-scrolling of the DMEK graft, once inside the anterior chamber, are indeed the most demanding steps in DMEK. The challenges occur as the DM, once detached from the cornea stromal surface, has an intrinsic propensity to adopt a scrolled configuration with the endothelial surface on its outside (46, 47). This is particularly the case for DMEK grafts harvested from young donors (46). Unlike conventional DSAEK, an alternative surgical skill set is needed by the corneal surgeon (42). The surgeon should understand the different described techniques to unscroll the DMEK graft once in the eye (48–50). Such techniques include methodological approaches to unfolding a double scrolled graft by tapping the cornea in a shallow anterior chamber, and the use of air bubbles to assist in tight or single scrolls (49, 50). In situations, for example tight scrolls or deep anterior chambers, the unscrolling of the graft can be technically demanding (46). Consequently, many corneal surgeons still reserve DMEK for more straightforward cases of endothelial diseases and DSAEK for more challenging cases (e.g., advanced bullous keratopathy, aphakia, large iris defects, vitrectomized eyes, previous glaucoma filtration surgery) (51–54).

In current clinical practice, the vast majority of DMEK surgeries performed are “endothelium-out” techniques. This was reflected in this systematic review. Of the 21,323 included eyes that underwent DMEK, 19,945 (93.5%) received their grafts through various “endothelium-out” insertion techniques. In these techniques, the DMEK graft is loaded into an injector and inserted into the anterior chamber as a scroll, with the endothelium on its outer surface. Injectors used included modified intraocular lens cartridges, implantable contact lens cartridges, intravenous tubing, or glass injectors (Supplementary Table 1). Direct contact of the endothelium of the DMEK graft to the walls of the injectors can potentially cause endothelial cell damage and loss. Studies have indicated that plastic graft injectors are associated with higher rates of post-operative graft detachments, compared to glass devices (55, 56). Such observations have been explained by more damage to the corneal endothelium with plastic materials, and intra-operative alterations in the morphologies of the grafts during insertion and un-scrolling, which may be caused by electro-static forces produced by plastic (55). Nonetheless, not all reports have found similar effects (57).

**FIGURE 5:**
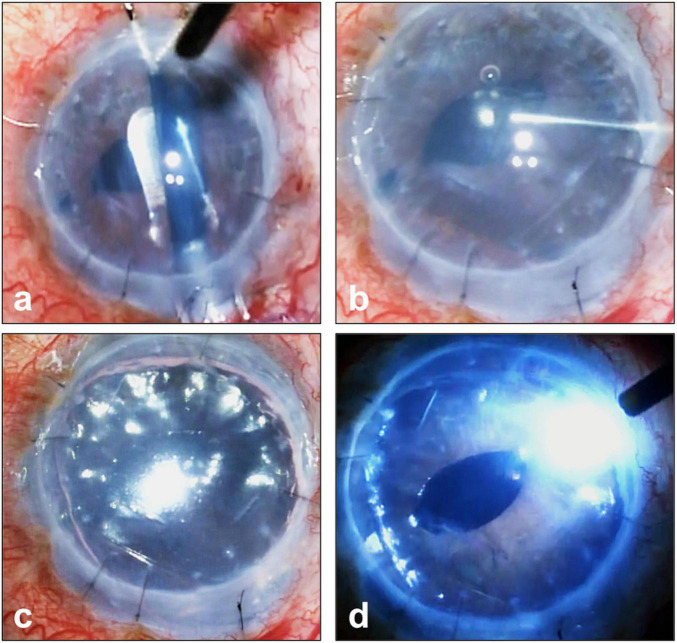
Complex Descemet membrane endothelial keratoplasty (DMEK) surgery performed in an eye with previously failed penetrating keratoplasty graft and iridocorneal endothelial (ICE) syndrome. **(a)** DMEK graft pre-stained with Membrane Blue Dual (D.O.R.C., Netherlands) and inserted into the eye. **(b)** Prolonged surgery has resulted in the loss of the blue stain making visualization of graft orientation and attachment difficult; **(c)** this is made more difficult given the patient’s dark iris **(d)** full air-gas tamponade of graft and the use of an external light pipe to assist the surgeon in graft orientation and attachment.

Moreover, in “endothelium-out” techniques, there is often no control of the scrolled graft during insertion. Despite the use of intraoperative imaging (58), orientation markers such as S-stamps (59) or other asymmetrical indicators (60), determining the orientation of the graft in the anterior chamber can sometimes be difficult. Especially in cases of prolonged surgery, DMEK grafts in the eye can lose their pre-stained trypan blue stains, making visualization of graft orientation even more difficult. This is especially so in patients with dark irides (Figure 5).

The unfolding of a scrolled DMEK graft and its central positioning on the recipient’s posterior stromal surface can also be problematic and time-consuming. To unfold the DMEK scroll after insertion into the anterior chamber, numerous approaches such as using air bubbles or jets of balanced salt solution in the presence of a shallow anterior chamber and the stroking of the corneal surface have been described (48). To overcome these difficulties of intracameral DMEK graft unfolding, different groups have investigated various alternative techniques. An example of such alternative techniques is the transplantation of DMEK tissue of various shapes (61). Authors have showed that certain DMEK graft shapes, such as the Maltese cross graft design, may be less prone to tight scrolling.

The concept of “endothelium-in” DMEK insertion techniques have been recently introduced (33–37, 44, 45). The grafts are folded, usually in a trifold, with the endothelium on the inside. These “endothelium-in” techniques prevent the DMEK grafts from adopting their natural scrolls with the endothelium on the outside. These “endothelium-in” techniques are believed to have the benefits of minimizing endothelial cell damage from the mechanical stress of the endothelial cells on the walls of the injectors. Moreover, in “endothelium-in” techniques, the grafts are pulled into the eyes with the endothelium facing downward. Once in the eye, the graft begins to unfold to acquire its physiological “endothelium-out” configuration, effectively “aiding” the surgeon in graft unfolding. Pre-clinical laboratory studies have also reported significantly shorter graft unfolding times for “endothelium-in” compared to “endothelium-out” techniques (62). These factors in “endothelium-in” techniques reduce the technical difficulties of intracameral graft orientation and unscrolling, making DMEK procedures more controlled and predictable. Some of these “endothelium-in” techniques also use devices created to mimic DSAEK techniques, which many corneal surgeons are accustomed to (33, 44, 45). Various laboratory studies have reported no significant differences in endothelial cell loss when DMEK grafts were loaded “endothelium-in” and pulled-through or loaded “endothelium-out” and injected-through different graft insertion devices (62–64). In this review, the surgical outcomes of both “endothelium-out” and “endothelium-in” techniques were evaluated.

### Summary of Evidence

This review included a total of 95 studies (Supplementary Table 1). Eighty-six studies using “endothelium-out” insertion techniques, eight studies using “endothelium-in” insertion techniques, and one study comparing “endothelium-out” to “endothelium-in” techniques. The majority of studies, 73/95 (76.8%), were rated as level III or level IV evidence. Only 4/95 (4.2%) studies were rated as level I evidence.

Evaluating the outcomes of “endothelium-out” techniques, the mean BCVA at 6 months after DMEK surgery ranged from 0.0 to 0.49 LogMAR (34 studies); 42.5–85% of eyes (15 studies) achieved a best-corrected visual acuity (BCVA) of 20/25 or better at 6 months. The mean endothelial cell loss ranged from 19 to 53%. The random pooled mean endothelial cell loss was 36.3 ± 6.4% at 6 months (27 studies) and 38.7 ± 7.2% at 12 months (12 studies). Rates of re-bubbling for graft detachments, primary graft failure rates, secondary graft failure rates, and graft rejection rates ranged from 0 to 82%, 0 to 21.0%, 0 to 7.0%, and 0 to 7.0%, respectively. The random pooled graft re-bubbling rates for *“endothelium-out”* techniques were 26.2% (95% CI 21.9–30.9%) (74 studies). The random pooled primary graft failure rates for *“endothelium-out”* techniques was 2.9% (95% CI 2.03–4.02%) (58 studies).

Of the eight “endothelium-in” studies reporting visual acuity data, the mean BCVA at 6 months after DMEK surgery ranged from 0.09 to 0.10 LogMAR (2 studies); 44.7–87.5% of eyes (3 studies) achieved a best-corrected visual acuity (BCVA) of 20/25 or better at 6 months. The mean endothelial cell loss ranged from 26.6 to 56.0% (7 studies). The random pooled mean endothelial cell loss was 28.1 ± 1.3% at 6 months (7 studies) and 29.6 ± 1.2% at 12 months (1 study). Graft detachment re-bubbling rates and primary graft failure rates ranged from 4.7 to 45.7% (nine studies) and 0 to 3.0% (six studies), respectively. Only one study reported on secondary graft failure rates in which there were none. None of the studies reporting on “endothelium-in” techniques reported the graft rejection rates. The random pooled graft re-bubbling rates for *“endothelium-in”* techniques was 16.5% (95% CI 8.5–26.4%) (6 studies). The random pooled primary graft failure rates for *“endothelium-in”* techniques was 1.5% (95% CI 0.6–2.7%) (six studies).

Comparing outcomes of *“endothelium-out”* to *“endothelium-in”* techniques, pooled mean endothelial cell loss was lower in the *“endothelium-in”* studies compared to *“endothelium-out”* studies at 6 months (*p* = 0.018); this was not statistically computable at 12 months as there was only 1 study for “*endothelium in”* group. Although re-bubbling rates for graft detachments were higher in the “endothelium-out” studies compared to “endothelium-in” studies, statistical significance was not achieved (*p* = 0.440). There was no significant difference in primary graft failure rates between the two groups (*p* = 0.552).

### Limitations of This Review

This review has several limitations. The quality of available evidence was considered low (grade III and IV) with a significant number of studies judged as having high risks of bias (Figure 3 and Supplementary Appendix 3). Significant heterogeneity existed in the studies, such as study designs, study population, surgical techniques, surgeon experience, outcome measures, and duration of follow-up. Studies published after the date of the pre-defined search strategy have also not been included. Furthermore, there was a smaller number of studies that reported on outcomes using “endothelium-in” DMEK surgeries that met the inclusion criteria for this review. This makes it difficult to provide any definitive conclusions through a comparative meta-analysis, especially in longer post-operative time points. Thus, the evidence to compare “endothelium-out” to “endothelium-in” techniques cannot be considered complete with this review.

## Conclusion

The rates of endothelial cell loss were reported to be significantly lower in “endothelium-in” DMEK surgeries at 6 months following surgery compared to “endothelium-out” surgeries. Despite the above-mentioned limitations, visual outcomes and rates of complications of “endothelium-in” techniques from the small number of studies were noted to be comparable to those reported in “endothelium-out” studies. Given the intra-operative challenges following graft insertion encountered using “endothelium-out” techniques, surgeons may consider “endothelium-in” techniques designed for easier intra-operative DMEK graft unfolding after graft insertion. However, further well-conducted, adequately powered, randomized controlled trials and studies with longer duration of follow-up are needed before conclusive comparisons between the two techniques can be made.

## Data Availability Statement

The original contributions presented in this study are included in the article/Supplementary Material, further inquiries can be directed to the corresponding authors.

## Author Contributions

HO and JM: conceptualization and supervision. HO and HH: data curation. HO, HH, MA, and JM: formal analysis, investigation, methodology, writing draft, and review and editing. All authors approved the manuscript.

## Conflict of Interest

JM holds a patent on the EndoGlide and receive royalties. The remaining authors declare that the research was conducted in the absence of any commercial or financial relationships that could be construed as a potential conflict of interest.

## Publisher’s Note

All claims expressed in this article are solely those of the authors and do not necessarily represent those of their affiliated organizations, or those of the publisher, the editors and the reviewers. Any product that may be evaluated in this article, or claim that may be made by its manufacturer, is not guaranteed or endorsed by the publisher.

## Footnotes

1
https://www.crd.york.ac.uk/PROSPERO

2
www.thecochranelibrary.com

3
www.clinicaltrials.gov

4
https://www.medcalc.org
