# Supplementary material for: “Endothelium-Out” and “Endothelium-In” Descemet Membrane Endothelial Keratoplasty (DMEK) Graft Insertion Techniques: A Systematic Review With Meta-Analysis
Source: Front Med (Lausanne). 2022 Jun 14;9:868533. doi: 10.3389/fmed.2022.868533 (PMC9237218; doi:10.3389/fmed.2022.868533)
Supplement: Supplementary file 1 [file Data_Sheet_2.docx]

**Supplementary Appendix 1: Protocol**

Submitted to PROSPERO International prospective register of systematic reviews (https://www.crd.york.ac.uk/PROSPERO; reference ID: 160657)

1. **Title**

**Endothelium-­­­­Out and Endothelium-In Descemet’s Membrane Endothelial Kerat­oplasty (DMEK) Graft Insertion Techniques: A Systematic Review**

*Hon Shing Ong^1,2,3^, Marcus Ang^1,3^, *Jodhbir S Mehta^1,2,3,4^

1. Corneal and External Diseases Department, Singapore National Eye Centre, Singapore
2. Tissue Engineering and Stem Cell Group, Singapore Eye Research Institute, Singapore
3. Duke-National University Singapore Graduate Medical School, Singapore
4. School of Material Science and Engineering, Nanyang Technological University, Singapore

*Corresponding authors:

Dr Ong Hon Shing / Professor Jodhbir S Mehta
Address: Singapore National Eye Centre, 11 Third Hospital Avenue, Singapore 168751

Telephone: +6563228378

E-mail: [honshing@gmail.com](mailto:honshing@gmail.com) / [jodmehta@gmail.com](mailto:jodmehta@gmail.com)

**Financial support**

None

**Conflict of interest**

J Mehta holds a patent on the EndoGlide and receive royalties. The authors have no other relevant affiliations or financial involvement with any organization or entity with a financial interest in or financial conflict with the subject matter or materials discussed in the manuscript apart from those disclosed.

1. **ABSTRACT**

The corneal endothelium is the innermost single cell-layer of the cornea. It plays an important role in the dynamic maintenance of corneal hydration, which is important for corneal clarity. Diseases of the corneal endothelium can result in an accelerated loss of corneal endothelial cells. In corneal endothelial failure, the cornea becomes oedematous, resulting in loss of corneal transparency and eventually blindness. Visual loss from corneal endothelial failure is a leading indication for corneal transplantation

In current practice, there are two main corneal transplantation techniques to treat corneal endothelial failure; Descemet’s stripping automated endothelial keratoplasty (DSAEK) and Descemet’s membrane endothelial keratoplasty (DMEK). DSAEK has been shown to achieve predictable visual outcomes and improved graft survival compared to older full thickness corneal transplantation techniques and thus has been widely adopted by corneal surgeons. DMEK, where only the Descemet’s membrane with its endothelium is harvested from donor tissue and transplanted, is the more recent advancement in corneal endothelial transplant surgery. DMEK has been shown to achieve more rapid visual recovery and better visual outcomes than DSAEK, in addition to a lower risk of transplant rejection.

However, many corneal surgeons have been slow to adopt DMEK as a predominant treatment for endothelial diseases. This has been attributed to the challenges in its surgical technique, and associated increased early complications, notably higher rates of graft detachment and iatrogenic graft failure due to inadvertent graft eversion. In the vast majority of techniques used in published studies, the DMEK graft is loaded and inserted such that its endothelium remains on the outside (‘endothelium-out’ techniques). More recently, the concept of ‘endothelium-in’ techniques for DMEK graft insertion has been described, introduced to make DMEK surgery easier and more predictable for the corneal surgeon by maintaining the orientation of the graft during insertion. Nevertheless, the differences in surgical outcomes of either technique for DMEK graft insertion, ‘endothelium-in’ or ‘endothelium-out’, remains unclear. This review aims to evaluate the published literature reporting the surgical outcomes of both ‘endothelium-out’ and ‘endothelium-in’ graft insertion techniques for DMEK.

**C. INTRODUCTION**

**1. Background**

**Description of condition**

Visual loss from corneal endothelial failure is a leading indication for corneal transplantation.^1,2^ Over the past two decades, the concept of selective replacement of damaged or lost endothelial cells using lamellar keratoplasty techniques has revolutionized the treatment of corneal endothelial failure.^3,4^ In the late 1990s, Melles first described an intrastromal approach for posterior lamellar keratoplasty.^5^ This selectively replaced only diseased corneal endothelium, and avoided full-thickness surgery. Subsequent modifications of this technique have since led to more advanced endothelial keratoplasty (EK) techniques with improved visual outcomes, lower risks of graft rejection, and superior graft survival rates.^6-8^ These EK techniques are minimally invasive, avoiding full-thickness central corneal trephination and intraoperative ‘open-sky’ situations, thereby reducing the risk of sight-threatening complications like expulsive haemorrhage. They also offer better mechanical integrity and overall globe strength. EK procedures have thus now replaced full-thickness penetrating keratoplasties (PK) as mainstay techniques for treating endothelial dysfunction.^1,2,9^

**Description of intervention**

In current practice, there are two leading techniques for EK performed worldwide: (i) Descemet’s stripping automated endothelial keratoplasty (DSAEK) or Descemet’s stripping endothelial keratoplasty (DSEK), depending on how the donor graft is prepared, and (ii) Descemet’s membrane endothelial keratoplasty (DMEK).^3,4,10^ In DSAEK/DSEK, transplanted donor endothelial grafts consist of donor endothelium, Descemet’s membrane (DM), and some posterior stroma. The development of devices for graft insertion has greatly simplified DSAEK/DSEK.^11^ Examples of such devices include glides such as EndoGlide (AngioTech, Reading, Pennsylvania, USA/Network Medical Products, North Yorkshire, UK) and the Busin glide (Moria Ophthalmic Instruments, Antony, France), or inserters such as the Endoserter (Ocular Systems, Winston-Salem, NC, USA). With predictable visual outcomes and improved graft survival compared to PK, DSAEK/DSEK has been widely adopted by corneal surgeons.^1^

DMEK is the more recent advancement in EK surgery.^12^ In DMEK, only the DM with its endothelium is harvested from donor tissue and transplanted. By only replacing tissues affected by endothelial diseases, DMEK is anatomically more accurate. As stromal tissue is not transplanted, DMEK avoids changes in corneal profiles, and is thus also associated with improved refractive outcomes. Indeed, DMEK has been shown to achieve more rapid visual recovery and better visual outcomes than DSAEK/DSEK.^13-17^ Studies comparing DMEK and DSAEK have also suggested lower endothelial rejection rates for DMEK.^18^

Current techniques of transferring the DMEK graft into the anterior chamber involve the insertion of the graft through a small corneal incision. Various instruments have been used for DMEK graft insertion. These include glass injectors^19,20^ and intraocular lens cartridges.^21,22^ All these instruments are designed to protect the DM scroll from the surgical wound. However, in the vast majority of techniques used in published studies, the DMEK graft is loaded such that its endothelium remains on the outside (‘endothelium-out’). The graft is thus potentially susceptible to endothelial cell loss caused by contact of its endothelium with the lumen of the injector. Furthermore, ‘endothelium-out’ techniques all involve the injection of the scroll of DMEK graft into the anterior chamber in its entirety. The unfolding of the free-floating graft, following its insertion, can be challenging, unpredictable, and time consuming.^23^ Such challenges have hindered corneal surgeons from adopting DMEK as a primary treatment for corneal endothelial failure.^1,3^ In a recent eye banking report, DSAEK/DSEK still accounted for over 75% of EK procedures performed in the United States.^1^

More recently, the concept of ‘endothelium-in’ techniques for DMEK graft insertion has been described.^24-27^ In these techniques, the harvested DM is folded and prevented from naturally scrolling with its endothelium on the outer surface. By maintaining the orientation of the DMEK graft during graft insertion, these ‘endothelium-in’ techniques aim to provide more control in graft unfolding following insertion into the eye.

**2. Rationale for this review**

Nevertheless, the differences in surgical outcomes of either technique for DMEK graft insertion, ‘endothelium-in’ or ‘endothelium-out’, remains unclear. This review aims to evaluate the published literature reporting the surgical outcomes of both ‘endothelium-out’ and ‘endothelium-in’ graft insertion techniques for DMEK. If the outcomes of ‘endothelium-in’ techniques were found to be comparable to those reported in ‘endothelium-out’ techniques, given the intra-operative challenges encountered with with the ‘endothelium-out’ techniques, surgeons may want to consider ‘endothelium-in’ techqniues, especially in eyes with more complex anterior segments. This may potentially increase the number of corneal surgeons adopting DMEK as a predominant treatment for endothelial diseases.

**3. Objectives of this review**

Hypothesis

We hypothesize that ‘endothelium-in’ techniques have comparable surgical outcomes to ‘endothelium-out’ graft insertion techniques for DMEK.

Aims

This review aims to evaluate the published literature reporting the surgical outcomes of both ‘endothelium-out’ and ‘endothelium-in’ graft insertion techniques for DMEK. Surgical outcomes include visual outcomes, endothelial cell loss, and complications including graft detachment / re-bubbling, graft rejection, and graft failure.

**D. METHODS**

**1. Eligibility Criteria for considering studies for this review**

**1.1 Types of Intervention**

We aim to include publications in which the surgical outcomes of DMEK performed for the treatment of endothelial dysfunction are reported.

**1.2 Types of Studies**

Study designs include controlled clinical trials, prospective or retrospective comparative observational studies, and large case series (≥ 25 eyes). Small case series (<25 eyes), letters, reviews, published abstracts, and laboratory-based studies will be excluded due to the high risk of bias in such reports.

**1.2 Types of Participants (study population)**

Studies reporting only surgical outcomes of DMEK performed for graft failure (including repeat DMEK surgery) or specific high-risk disease groups (e.g. glaucoma, cytomegalovirus endotheliitis, herpes simplex) will also be excluded. To avoid duplicate reporting of similar study populations, where the same group of investigators published several studies, earlier smaller studies will be excluded if more recent larger studies reporting the same outcome measures are available.

**2.** **Information sources**

Information sources will include all applicable electronic databases, all relevant articles in the reference list of any relevant article, and all relevant articles which cite any relevant article.

**3. Search methods for identification of studies**

Electronic literature searches will be conducted in the following databases: CENTRAL, Cochrane Library databases (www.thecochranelibrary.com), PubMed, EMBASE, ClinicalTrials.gov (www.clinicaltrials.gov). No date or language restrictions will be set in our electronic searches. Key search terms will include the MeSH headings Descemet membrane endothelial keratoplasty, Descemet’s membrane endothelial keratoplasty, and DMEK.

*Searching other resources*

We will search the reference lists of the studies included in the review for information about further clinical studies. We will contact authors if further information is required.

**4. Data collection and analysis**

**4.1 Selection of studies**

The titles and abstracts resulting from the searches will be examined. Duplicate studies and clearly irrelevant studies will be removed. Full-text copies of potentially relevant reports will be retrieved and these will be assessed against our criteria for considering studies for this review.

**4.2 Data extraction and management**

Only data from eyes that had received DMEK surgeries will be included. Where studies reported on the outcomes of eyes that had undergone surgeries other than DMEK, these eyes will be excluded from the review. The following details of each study will be extracted for this review: study participants’ characteristics, study design, DMEK graft insertion techniques, and surgical outcome measures.

**4.3 Assessment of risks of bias in included studies**

The study design of each article will be assessed and rated according to its level of evidence. A rating scale adapted from the Oxford Centre for Evidence-based Medicine will be used.^28^ Studies that meet the inclusion criteria will also assessed for risk of bias using Chapter 8 of the *Cochrane Handbook for Systematic Reviews of Intervention.*^29^ The following domains for potential risk of bias will be considered: a) *selection bias* - random sequence generation (to determine whether the sequence allocation was adequately generated), b) *selection bias* - allocation concealment, c) *performance / detection bias* - masking of outcome assessors and participants (to determine whether knowledge of the allocated intervention was adequately prevented during the study), d) *attrition bias* incomplete outcome data, and e) *reporting bias* - selective outcome reporting. We will grade each domain of trial as ‘low risk’ of bias, ‘high risk’ of bias, or ‘unclear risk’. The assessments will also consider the risk of material bias. We define material bias as bias of sufficient magnitude to have a notable impact on the results or conclusions of the trial, recognizing that subjectivity is involved in any such judgement. We will resolve any disagreements between the review authors by discussion.

**4.4 Summary measures**

Data on the following surgical outcome measures will be obtained: visual outcomes, endothelial cell loss, and complications including graft detachment / re-bubbling, graft rejection, and graft failure. For direct comparison of visual outcomes, logarithm of the minimum angle of resolution (LogMAR) measures of visual acuities will be converted to the respective Snellen VA equivalents.

**4.5 Measures of treatment effect**

The preferred unit of analysis will be outcomes for eyes rather than individuals, since some individuals will have unilateral treatment or different treatments in each eye. We will include paired-eye studies, where one eye received one intervention and the second eye had gone on to receive another intervention. Similarly, we will include studies where both eyes received the same intervention.

**4.6 Dealing with missing data**

Where we are unable to extract all the information we are interested in from published reports, both with regard to the details of the study and its numerical results, we aim to request the missing data from the original investigators.

**4.7 Data synthesis**

We will perform data analysis according to Chapter 9 of the Cochrane Handbook for Systematic Reviews of Interventions.^29^ Depending on the studies that will be included, we will perform a meta-analysis if this is appropriate. If a meta-analysis is appropriate, we will use a fixed-effect or random-effects model depending on the include study.

In the event that a meta-analysis cannot be performed due to the lack of quantitative data, narrative synthesis regarding the direction of effect, size of effect, consistency of effect across the studies and the strength of evidence will be presented.

**4.8 Assessment of risk of bias across studies**

For each study, we will assess the risk of bias that may affect the cumulative evidence (such as publication bias, selective reporting within studies). If a protocol of an individual study is available, the protocol and the published results will be compared. In the absence of a protocol, outcomes measures listed in the methodology section of the published report will be compared to those in the published results.

**4.9 Assessment of heterogeneity**

We endeavor to identify differences between the studies which are likely to introduce heterogeneity. As some degree of heterogeneity always exists due to the clinical and methodological diversity of the studies, where appropriate, we will employ the results of the Chi^2^ test as well as I^2^ statistic to quantify inconsistencies across the studies.

**E. LIMITATIONS**

We anticipate that significant heterogeneity exists in studies that are currently in the literature and that the majority of studies will not be well-conducted randomized controlled studies. Thus the quality of evidence will thus be considered low. Furthermore, there may be only a small number of studies using ‘endothelium-in’ techniques meeting our inclusion criteria. The evidence to compare ‘endothelium-out’ and ‘endothelium-in’ techniques thus may not be considered complete even after this review.

**CONTRIBUTIONS**

HSO and JSM conceived the review question. HSO will perform the electronic searches. HSO and JSM will be involved in the extraction and interpretation of the data. HSO, MA, and JSM will be involved in drafting the manuscript for publication.

**REFERENCES**

1. EBAA. EYE BANKING STATISTICAL REPORT. 2016. <http://restoresight.org/wp-content/uploads/2017/04/2016_Statistical_Report-Final-040717.pdf> (accessed 18/02/2019.

2. Australian Corneal Graft Registry C. THE AUSTRALIAN GRAFT REGISTRY 2018 REPORT. 2018. [https://dspace.flinders.edu.au/xmlui/bitstream/handle/2328/37917/ACGR 2018 Report.pdf?sequence=3&isAllowed=y](https://dspace.flinders.edu.au/xmlui/bitstream/handle/2328/37917/ACGR%202018%20Report.pdf?sequence=3&isAllowed=y) (accessed 09/05/2019.

3. Park CY, Lee JK, Gore PK, Lim CY, Chuck RS. Keratoplasty in the United States: A 10-Year Review from 2005 through 2014. *Ophthalmology* 2015; **122**(12): 2432-42.

4. Tan DT, Dart JK, Holland EJ, Kinoshita S. Corneal transplantation. *Lancet* 2012; **379**(9827): 1749-61.

5. Melles GR, Eggink FA, Lander F, et al. A surgical technique for posterior lamellar keratoplasty. *Cornea* 1998; **17**(6): 618-26.

6. Koenig SB, Covert DJ, Dupps WJ, Jr., Meisler DM. Visual acuity, refractive error, and endothelial cell density six months after Descemet stripping and automated endothelial keratoplasty (DSAEK). *Cornea* 2007; **26**(6): 670-4.

7. Ang M, Soh Y, Htoon HM, Mehta JS, Tan D. Five-Year Graft Survival Comparing Descemet Stripping Automated Endothelial Keratoplasty and Penetrating Keratoplasty. *Ophthalmology* 2016; **123**(8): 1646-52.

8. Woo JH, Ang M, Htoon HM, Tan DT. Descemet Membrane Endothelial Keratoplasty versus Descemet Stripping Automated Endothelial Keratoplasty and Penetrating Keratoplasty. *Am J Ophthalmol* 2019.

9. Guell JL, El Husseiny MA, Manero F, Gris O, Elies D. Historical Review and Update of Surgical Treatment for Corneal Endothelial Diseases. *Ophthalmol Ther* 2014; **3**(1-2): 1-15.

10. Price FW, Jr., Feng MT, Price MO. Evolution of Endothelial Keratoplasty: Where Are We Headed? *Cornea* 2015; **34 Suppl 10**: S41-7.

11. Ang M, Saroj L, Htoon HM, Kiew S, Mehta JS, Tan D. Comparison of a donor insertion device to sheets glide in Descemet stripping endothelial keratoplasty: 3-year outcomes. *Am J Ophthalmol* 2014; **157**(6): 1163-9 e3.

12. Melles GR, Ong TS, Ververs B, van der Wees J. Descemet membrane endothelial keratoplasty (DMEK). *Cornea* 2006; **25**(8): 987-90.

13. Singh A, Zarei-Ghanavati M, Avadhanam V, Liu C. Systematic Review and Meta-Analysis of Clinical Outcomes of Descemet Membrane Endothelial Keratoplasty Versus Descemet Stripping Endothelial Keratoplasty/Descemet Stripping Automated Endothelial Keratoplasty. *Cornea* 2017; **36**(11): 1437-43.

14. Droutsas K, Lazaridis A, Papaconstantinou D, et al. Visual Outcomes After Descemet Membrane Endothelial Keratoplasty Versus Descemet Stripping Automated Endothelial Keratoplasty-Comparison of Specific Matched Pairs. *Cornea* 2016; **35**(6): 765-71.

15. Tourtas T, Laaser K, Bachmann BO, Cursiefen C, Kruse FE. Descemet membrane endothelial keratoplasty versus descemet stripping automated endothelial keratoplasty. *Am J Ophthalmol* 2012; **153**(6): 1082-90 e2.

16. Guerra FP, Anshu A, Price MO, Price FW. Endothelial keratoplasty: fellow eyes comparison of Descemet stripping automated endothelial keratoplasty and Descemet membrane endothelial keratoplasty. *Cornea* 2011; **30**(12): 1382-6.

17. Stuart AJ, Romano V, Virgili G, Shortt AJ. Descemet's membrane endothelial keratoplasty (DMEK) versus Descemet's stripping automated endothelial keratoplasty (DSAEK) for corneal endothelial failure. *Cochrane Database Syst Rev* 2018; **6**: CD012097.

18. Marques RE, Guerra PS, Sousa DC, Goncalves AI, Quintas AM, Rodrigues W. DMEK versus DSAEK for Fuchs' endothelial dystrophy: A meta-analysis. *Eur J Ophthalmol* 2018: 1120672118757431.

19. Dapena I, Moutsouris K, Droutsas K, Ham L, van Dijk K, Melles GR. Standardized "no-touch" technique for descemet membrane endothelial keratoplasty. *Arch Ophthalmol* 2011; **129**(1): 88-94.

20. Arnalich-Montiel F, Munoz-Negrete FJ, De Miguel MP. Double port injector device to reduce endothelial damage in DMEK. *Eye (Lond)* 2014; **28**(6): 748-51.

21. Kruse FE, Laaser K, Cursiefen C, et al. A stepwise approach to donor preparation and insertion increases safety and outcome of Descemet membrane endothelial keratoplasty. *Cornea* 2011; **30**(5): 580-7.

22. Kim EC, Bonfadini G, Todd L, Zhu A, Jun AS. Simple, inexpensive, and effective injector for descemet membrane endothelial keratoplasty. *Cornea* 2014; **33**(6): 649-52.

23. Maier AK, Gundlach E, Schroeter J, et al. Influence of the difficulty of graft unfolding and attachment on the outcome in Descemet membrane endothelial keratoplasty. *Graefes Arch Clin Exp Ophthalmol* 2015; **253**(6): 895-900.

24. Ang M, Mehta JS, Newman SD, Han SB, Chai J, Tan D. Descemet Membrane Endothelial Keratoplasty: Preliminary Results of a Donor Insertion Pull-through Technique Using a Donor Mat Device. *Am J Ophthalmol* 2016; **171**: 27-34.

25. Busin M, Leon P, D'Angelo S, et al. Clinical Outcomes of Preloaded Descemet Membrane Endothelial Keratoplasty Grafts With Endothelium Tri-Folded Inwards. *Am J Ophthalmol* 2018; **193**: 106-13.

26. Leon P, Parekh M, Nahum Y, et al. Factors Associated With Early Graft Detachment in Primary Descemet Membrane Endothelial Keratoplasty. *Am J Ophthalmol* 2018; **187**: 117-24.

27. Price MO, Lisek M, Kelley M, Feng MT, Price FW, Jr. Endothelium-in Versus Endothelium-out Insertion With Descemet Membrane Endothelial Keratoplasty. *Cornea* 2018; **37**(9): 1098-101.

28. CEBM. Oxford Centre for Evidence-based Medicine – Levels of Evidence. 2009. <https://www.cebm.net/2009/06/oxford-centre-evidence-based-medicine-levels-evidence-march-2009/> (accessed 18/02/2019.

29. Higgins JPT, Altman DG, Sterne JAC. Chapter 8: Assessing risk of bias in included studies In: Higgins JPT, Green S (editors). Cochrane Handbook for Systematic Reviews of Interventions Version 5.1.0 (updated March 2011). The Cochrane Collaboration. 2011. <www.cochrane-handbook.org>.
