# Supplementary material for: “Endothelium-Out” and “Endothelium-In” Descemet Membrane Endothelial Keratoplasty (DMEK) Graft Insertion Techniques: A Systematic Review With Meta-Analysis
Source: Front Med (Lausanne). 2022 Jun 14;9:868533. doi: 10.3389/fmed.2022.868533 (PMC9237218; doi:10.3389/fmed.2022.868533)
Supplement: Supplementary file 2 [file Data_Sheet_1.DOCX]

**Supplementary Appendix 2: SEARCH STRATEGIES**

- CENTRAL search strategy

#1 MeSH descriptor: [Descemet membrane endothelial keratoplasty]

#2 MeSH descriptor: [Descemet’s membrane endothelial keratoplasty]

#3 MeSH descriptor: [DMEK]

#4 #1 or #2 or #3

- PubMed search strategy

DMEK[All Fields] OR (("descemet membrane"[MeSH Terms] OR ("descemet"[All Fields] AND "membrane"[All Fields]) OR "descemet membrane"[All Fields]) AND ("endothelium"[MeSH Terms] OR "endothelium"[All Fields] OR "endothelial"[All Fields]) AND ("corneal transplantation"[MeSH Terms] OR ("corneal"[All Fields] AND "transplantation"[All Fields]) OR "corneal transplantation"[All Fields] OR "keratoplasty"[All Fields])) OR (("descemet membrane"[MeSH Terms] OR ("descemet"[All Fields] AND "membrane"[All Fields]) OR "descemet membrane"[All Fields] OR ("descemet's"[All Fields] AND "membrane"[All Fields]) OR "descemet's membrane"[All Fields]) AND ("endothelium"[MeSH Terms] OR "endothelium"[All Fields] OR "endothelial"[All Fields]) AND ("corneal transplantation"[MeSH Terms] OR ("corneal"[All Fields] AND "transplantation"[All Fields]) OR "corneal transplantation"[All Fields] OR "keratoplasty"[All Fields]))

- EMBASE (OvidSP) search strategy

(Descemet membrane endothelial keratoplasty or Descemet's membrane endothelial keratoplasty or DMEK).mp. [mp=title, abstract, heading word, drug trade name, original title, device manufacturer, drug manufacturer, device trade name, keyword, floating subheading word, candidate term word]

- ClinicalTrials.gov search strategy

Descemet membrane endothelial keratoplasty OR Descemet’s membrane endothelial keratoplasty OR DMEK
