# Supplementary material for: “Endothelium-Out” and “Endothelium-In” Descemet Membrane Endothelial Keratoplasty (DMEK) Graft Insertion Techniques: A Systematic Review With Meta-Analysis
Source: Front Med (Lausanne). 2022 Jun 14;9:868533. doi: 10.3389/fmed.2022.868533 (PMC9237218; doi:10.3389/fmed.2022.868533)
Supplement: Supplementary file 3 [file Data_Sheet_3.DOCX]

Supplementary Appendix 3. Authors’ judgements of each risk of bias item for each included study.

| **Study** | **Random sequence generation (selection bias)** | **Allocation concealment (selection bias)** | **Blinding of participants and personnel (performance bias)** | **Blinding of outcome assessment (detection bias)** | **Incomplete outcome data (attrition bias)** | **Selective reporting (reporting bias)** |
| --- | --- | --- | --- | --- | --- | --- |
| Price 2014 | L | L | H | H | L | U |
| Price 2015 | L | L | H | H | L | U |
| Chamberlain 2019 | L | H | L | L | L | U |
| Dunker 2020 | L | L | L | H | L | U |
| Santander-García 2019 | L | L | H | L | L | U |
| Price 2009 | H | H | H | H | L | U |
| Rudolph 2012 | H | H | H | H | U | H |
| Tourtas 2012 | H | H | H | H | H | U |
| Feng 2013 | H | H | H | H | L | U |
| Chaurasia 2014 | H | H | H | H | H | H |
| Cabrerizo 2014 | H | H | H | H | L | U |
| Guell 2015 | H | H | H | H | L | H |
| Hamzaoglu 2015 | H | H | H | H | H | H |
| Heinzelmann 2015 | H | H | H | H | L | H |
| Veldman 2016 | H | H | H | H | H | H |
| Droutsas 2016 | H | H | H | H | L | H |
| Heinzelmann 2016 | H | H | H | H | U | U |
| Price 2016 | H | H | H | H | L | U |
| Schaub 2017 | H | H | H | H | L | H |
| Philips 2017 | H | H | H | H | U | H |
| Aravena 2017 | H | H | H | H | L | H |
| Tourtas 2014 | H | H | H | H | L | H |
| Gundlach 2015 | H | H | H | H | L | H |
| Röck 2012 | H | H | H | H | L | H |
| Maier 2015 | H | H | H | H | L | U |
| Hoerster 2016 | H | H | H | H | L | U |
| Schaub 2017 | H | H | H | H | H | H |
| Reigner 2017 | H | H | H | H | L | H |
| Botsford 2016 | H | H | H | H | L | H |
| Rickmann 2018 | H | H | H | H | U | H |
| Schrittenloher 2018 | H | H | H | H | U | H |
| Brockmann 2018 | H | H | H | H | H | U |
| Koçluk 2018 | H | H | H | H | U | H |
| Rickmann 2018 | H | H | H | H | L | H |
| Von Marchtaler 2018 | H | H | H | H | L | H |
| Rickmann 2019 | H | H | H | H | L | U |
| Shahnazaryan 2020 | H | H | H | H | L | U |
| Koechel 2020 | H | H | H | H | L | U |
| Potts 2020 | H | H | H | H | L | U |
| Böhm 2021 | H | H | H | H | L | H |
| Zwingelberg 2021 | H | H | H | H | L | U |
| Laaser 2012 | H | H | H | H | H | U |
| Jansen 2021 | H | H | H | H | L | U |
| Guerra 2011 | H | H | H | H | H | U |
| Laaser 2012 | H | H | H | H | U | H |
| Parker 2012 | H | H | H | H | L | H |
| Anshu 2012 | H | H | H | H | H | H |
| Gorovoy 2014 | H | H | H | H | H | H |
| Monnereau 2014 | H | H | H | H | H | H |
| Burkhart 2014 | H | H | H | H | L | H |
| Maier 2014 | H | H | H | H | H | H |
| Feng 2014 | H | H | H | H | H | H |
| Deng 2015 | H | H | H | H | L | H |
| Rodríguez-Calvo-de-Mora 2015 | H | H | H | H | H | H |
| Bhandari 2015 | H | H | H | H | H | H |
| Schoenberg 2015 | H | H | H | H | H | H |
| Gorovoy 2015 | H | H | H | H | L | H |
| Ham 2016 | H | H | H | H | H | H |
| Siggel 2016 | H | H | H | H | U | H |
| van Dijk 2016 | H | H | H | H | H | U |
| Schlögl 2016 | H | H | H | H | H | H |
| Rose-Nussbaumer 2016 | H | H | H | H | L | U |
| Bhandari 2016 | H | H | H | H | L | U |
| Debellemaniere 2017 | H | H | H | H | U | U |
| Oellerich 2017 | H | H | H | H | H | H |
| Peraza-Nieves 2017 | H | H | H | H | H | U |
| Showail 2018 | H | H | H | H | L | H |
| Heinzelmann 2018 | H | H | H | H | L | U |
| Basak 2018 | H | H | H | H | L | U |
| Kurji 2018 | H | H | H | H | L | U |
| Fajgenbaum 2018 | H | H | H | H | H | H |
| Newman 2018 | H | H | H | H | H | U |
| Price 2018 | H | H | H | H | H | H |
| Schrittenlocher 2018 | H | H | H | H | L | H |
| Droutsas 2018 | H | H | H | H | H | H |
| Godin 2019 | H | H | H | H | L | H |
| Rickmann 2019 | H | H | H | H | H | H |
| Sarnicola 2019 | H | H | H | H | H | H |
| Brockmann 2019 | H | H | H | H | U | U |
| Schaub 2019 | H | H | H | H | U | H |
| Livny 2019 | H | H | H | H | L | H |
| Basak 2020 | H | H | H | H | L | U |
| Siddharthan 2020 | H | H | H | H | L | U |
| Lekhanont 2021 | H | H | H | H | L | H |
| Marchand 2021 | H | H | H | H | L | H |
| Studney 2021 | H | H | H | H | L | U |
| Ang 2016 | H | H | H | H | L | U |
| Busin 2018 | H | H | H | H | H | U |
| Leon 2018 | H | H | H | H | L | H |
| Tan 2020 | H | H | H | H | L | U |
| Yu 2020 | H | H | H | H | L | U |
| Woo 2020 | H | H | H | H | L | H |
| Jabbour 2021 | H | H | H | H | L | U |
| Ighani 2021 | H | H | H | H | L | H |
| Price 2018 | H | H | H | H | L | H |

H = High risk; L = Low risk; U = Unclear risk
