# Supplementary material for: “Endothelium-Out” and “Endothelium-In” Descemet Membrane Endothelial Keratoplasty (DMEK) Graft Insertion Techniques: A Systematic Review With Meta-Analysis
Source: Front Med (Lausanne). 2022 Jun 14;9:868533. doi: 10.3389/fmed.2022.868533 (PMC9237218; doi:10.3389/fmed.2022.868533)
Supplement: Supplementary file 4 [file Table_1.docx]

**Supplementary Table 1 Summary of included studies**

| **Author(s), Year** | **Level of Evidence** | **Number of Eyes (n)** | **Follow-up (months)** | **Method / device of graft insertion** | **DMEK subgroups (n)** | **Reported post-operative mean BCVA**^‡^ | **Mean Endothelial cell loss (%)** | **Re-bubbling rate (%)** | **Primary graft failure rate (%)** | **Secondary graft failure (%)** | **Immune rejection rate (%)** | **Other complications** |
| --- | --- | --- | --- | --- | --- | --- | --- | --- | --- | --- | --- | --- |
| **Endothelium out techniques** | | | | | | | | | | | | |
| Price et al,^63^ 2014 | 1 | 325 | 12 | ICL injector and IOL injector | PF (164) |  | 30^*^  (IQR 21-29) |  |  |  | 0 | Raised IOP (21.9%)^†^ |
|  |  |  |  |  | FML (161) |  | 31^*^  (IQR 20-41) |  |  |  | 1.4 | Raised IOP (6.1%)^†^;  CME (0.6%) |
| Price et al,^64^ 2015 | 1 | 232 | 12 | ICL injector | PF (116) |  | 29 ± 12 |  |  |  | 0 | Raised IOP (25.0%); corneal ulcer (0.9%) |
|  |  |  |  |  | LP (116) |  | 32 ± 15 |  |  |  | 0 | Raised IOP (11.0%) |
| Chamberlain et al,^65^ 2019 | 1 | 25 | 12 | Glass injector | DMEK (25) | 0.04 ± 0.12 (20/20) |  | 24.0 | 4.0 |  | 0 |  |
| Dunker et al,^66^ 2020 | 1 | 29 | 12 | Glass injector | DMEK (29) | 0.08 ± 0.14 | 30.2 | 24.1 |  |  | 0 | Raised IOP (17.2%); CME (3.4%) |
| Santander-García D et al;^67^ 2019 | 2 | 117 | 6 | Glass injector | DMEK (117) |  | 41 ± 17.0  (30.7^†^) | 11.1 | 0 | 0 | 0 |  |
| Price et al,^68^ 2009 | 2 | 60 | 3 | ICL injector and IOL injector | DMEK (60) | 20/25; 26% = 20/20; 63% ≥ 20/25; 94% ≥20/40 | 30 ± 20 | 63 | 8.0 |  | 3.0 |  |
| Rudolph et al,^69^ 2012 | 2 | 30 | 6.5 | IOL injector | DMEK (30) | 0.16 ± 0.08 |  |  |  |  |  |  |
| Tourtas et al,^23^ 2012 | 2 | 38 | 6 | IOL injector | DMEK (38) | 0.17 ± 0.12 | 41 | 82 | 0 |  |  |  |
| Feng et al,^70^ 2013 | 2 | 361 | 3 | ICL injector | Donor prepared on day of surgery (130) |  | 28^*^ | 15 | 1.5 |  |  |  |
|  |  |  |  |  | Donor prepared 1 day ahead (160) |  | 29^*^ | 13 | 1.9 |  |  |  |
|  |  |  |  |  | Donor prepared 2 days ahead (71) |  | 29^*^ | 14 | 2.8 |  |  |  |
| Chaurasia et al,^71^ 2014 | 2 | 423 | 6 | ICL injector | DMEK (243) | 0.10 (20/25) | 27 | 30 | 3.1 |  | 0 | CME (1%) |
|  |  |  |  |  | DMEK + CS (180) | 0.0 (20/20) | 25 | 29 | 3.5 |  | 0 | CME (1.5%) |
| Cabrerizo et al,^72^ 2014 | 2 | 29 | 7.3 | Glass injector | Phakic DMEK (12) | 0.05 ± 0.70 |  |  |  |  |  |  |
|  |  |  |  |  | Pseudophakic DMEK (17) | 0.02 ± 0.70 |  |  |  |  |  |  |
| Guell et al,^73^ 2015 | 2 | 81 | 36 | IOL injector | 20% SF_6_ (42) | 0.04 ± 0.23 | 30.4 ± 11 | 2.4 | 2.4 | 0 | 0 |  |
|  |  |  |  |  | 100% Air (39) | 0.09 ± 0.13 | 32.6 ± 13 | 12.8 | 0 | 0 | 0 |  |
| Hamzaoglu,^41^ 2015 | 2 | 100 | 6 | Glass injector | DMEK (100) | 0.11±0.13 | 27.9 ± 16.0 | 6.0 | 4.0 | 0 | 0 |  |
| Heinzelmann et al,^74^ 2015 | 2 | 155 | 6 | IOL injector | DMEK (80) |  |  | 18 | 1.3 |  |  | CME (12.5%) |
|  |  |  |  |  | DMEK + CS (75) |  |  | 23 | 4.0 |  |  | CME (13.3%) |
| Veldman et al,^57^ 2016 | 2 | 165 | 6 | Glass injector | Unstamped (32) | 0.11 ± 0.13 | 29 ± 14 | 3 | 12.5 | 0 | 3.1 |  |
|  |  |  |  |  | S-stamped (133) | 0.10 ± 0.14 | 31 ± 17 | 13 | 0.8 | 0 | 0.8 |  |
| Droutsas et al,^22^ 2016 | 2 | 25 | 12 | Laboratory glass pipette | DMEK (25) | 0.0^*^ (range, -0.08 to 0.7) | 44.2^*^ |  |  |  |  |  |
| Heinzelmann et al,^75^ 2016 | 2 | 450 | 42 | IOL injector | DMEK (450) | ≥20/25 (53%) |  | 20 |  | 7.0 | 7.0 |  |
| Price et al,^40^ 2016 | 2 | 400 | 24 | ICL injector | Corticosteroid  after 1 year (123) |  | -5.6 ± 12 /year |  |  | 0 | 0 | Raised IOP (1.6%) |
|  |  |  |  |  | No corticosteroid after 1 year (277) |  | -6.4 ± 14/year |  |  | 0.4 | 6 | Raised IOP (0.04%) |
| Schaub et al,^76^ 2017 | 2 | 854 | 12 | IOL injector | 20% SF_6_ (104) | 0.11 ± 0.10 | 35.8 | 23.8 | 0 | 2.9 | 0 |  |
|  |  |  |  |  | 100% Air (749) | 0.16 ± 0.15 | 38.1 | 53.9 | 0.1 | 2.3 | 0.3 |  |
| Philips et al,^42^ 2017 | 2 | 100 | 6 | Glass injector | DMEK (100) | 0.0844; 54.5% ≥ 20/20; 97% ≥ 20/40 | 31.9 | 5.0 | 1.0 | 0 |  | Posterior synechiae (5%) |
| Aravena et al,^77^ 2017 | 2 | 60 | 3 | IOL injector | DMEK  (No glaucoma) | 20/25 | 32.7 | 23.3 | 1.6 | 0 | 0.9 | Raised IOP (23.3%) |
| Tourtas et al,^78^ 2014 | 3 | 53 | 1 | IOL injector | 10mm descemetorhexis |  |  | 6.7  (33.3^†^) |  |  |  |  |
|  |  |  |  |  | 6mm descemetorhexis |  |  | 30.4  (78.3^†^) |  |  |  |  |
| Gundlach et al,^79^ 2015 | 3 | 67 | 6 | Glass injector | Phakic DMEK (13) | 0.13 ± 0.12; ≥20/25 (55.0%); ≥20/20 (36.0%) | 34 | 18^†^ |  |  | 0 | Raised IOP (15.4%); cataract progression (15.4%) |
|  |  |  |  |  | DMEK + CS (54) | 0.15 ± 0.11; ≥20/25 (52.0%); ≥20/20 (17.0%) | 27 | 50^†^ |  |  | 1.9 | Raised IOP (7.4%) |
| Röck et al,^80^ 2015 | 3 | 160 | 13.9 | Glass injector | Centred graft (115) | 0.12 ± 0.11 | 31.6 | 15 |  |  |  |  |
|  |  |  |  |  | Decentred graft (45) | 0.23  ±  0.29 | 35.5 | 44 |  |  |  |  |
| Maier et al,^31^ 2015 | 3 | 169 | 17.7 | Glass injector | Straightforward (63) | 0.21  ± 0.25 | 21.0 | 37.0 | 0 | 0 | 0.9 | CME (0.8%) |
|  |  |  |  |  | Manipulation <5min (47) | 0.19  ± 0.20 | 24.0 | 44.7 | 0 | 0 | 0 |  |
|  |  |  |  |  | Manipulation >5min + repeat air exchange (32) | 0.21 ± 0.27 | 23.0 | 43.8 | 0 | 0 | 0 |  |
|  |  |  |  |  | Direct manipulation of graft (18) | 0.27 ± 0.32 | 42.0 | 50.0 | 0 | 0 | 0 |  |
| Hoerster et al,^81^ 2016 | 3 | 150 | 6 | IOL injector | Intense PF (75) | 0.23 ± 0.16 | 38 | 67 |  |  |  | Raised IOP (2.7%); CME (0%) |
|  |  |  |  |  | Standard PF (75) | 0.22 ± 0.22 | 38 | 52 |  |  |  | Raised IOP (0%);  CME (12%) |
| Schaub et al,^82^ 2017 | 3 | 181 | 24 | IOL injector | Phakic donors (136) |  | 39 | 51.5 | 0 | 5.1 | 2.2 |  |
|  |  |  |  |  | Pseudophakic donors (45) |  | 38 | 60 | 2.2 | 6.7 | 0 |  |
| Regnier et al,^83^ 2017 | 3 | 33 | 6 | Glass injector | EB prepared (11) | 0.10^*^(IQR 0.00–0.20) | 41.0 | 45.5 | 9.1 |  | 0 | CME (9.1) |
|  |  |  |  |  | Surgeon prepared (22) | 0.10^*^ (IQR 0.10–0.30) | 46.0 | 68.2 | 18.2 |  | 0 | CME (13.6) |
| Botsford et al^84^ 2016 | 3 | 42 | 6 | IOL cartridge | 20% SF_6_ (21) | 0.09 ± 0.10; ≥20/25 (71.0%) |  | 19 | 0 | 0 | 0 |  |
|  |  |  |  |  | 100% Air (21) | 0.10 ± 0.12; ≥20/25 (67.0%) |  | 67 | 0 | 0 | 0 |  |
| Rickmann et al,^85^ 2018 | 3 | 200 | 13.7 | Glass injector | DMEK (200) | 0.25 ± 0.29 | 29.9 | 22.0 |  |  |  |  |
| Schrittenloher et al,^86^ 2018 | 3 | 693 | 12 | IOL injector | 8mm graft | 0.12 ± 0.08 | 36 ± 16 | 26.3 |  |  |  |  |
|  |  |  |  |  | 10mm graft | 0.09 ± 0.07 | 38 ± 14 | 59.6 |  |  |  |  |
| Brockmann et al,^87^ 2018 | 3 | 119 | 12 | Glass injector | FECD (83) | 0.10 ± 0.09 | 23.7 | 40.6 |  |  |  |  |
|  |  |  |  |  | BK (14) | 0.15 ± 0.11 | 36.9 | 38.9 |  |  |  |  |
| Koçluk et al,^88^ 2018 | 3 | 50 | 6 | Glass injector | First 25 (25) | 0.49 ± 0.51 |  | 4.0 | 12.0 |  |  |  |
|  |  |  |  |  | Subsequent 25 (25) | 0.47 ± 0.52 |  | 4.0 | 0 |  |  |  |
| Rickmann et al,^89^ 2018 | 3 | 108 | 6 | Glass injector | 20% SF_6_ (54) | 0.21 ± 0.29 | 31.0 | 22.2 | 5.6 |  | 0 |  |
|  |  |  |  |  | 100% Air (54) | 0.38 ± 0.33 | 34.8 | 18.5 | 3.7 |  | 0 |  |
| Von Marchtaler et al,^90^ 2018 | 3 | 136 | 3 | IOL injector | 20% SF_6_ (68) | 0.22 ± 0.16 | 39.6 | 8.8 |  |  |  |  |
|  |  |  |  |  | 100% Air (68) | 0.25 ± 0.15 | 37.3 | 33.8 |  |  |  |  |
| Rickmann et al,^91^ 2019 | 3 | 51 | 6 | Glass injector | Precut DMEK (22) | 0.2 ± 0.14 | 34.0 | 18.0 | 9.1 |  | 0 |  |
|  |  |  |  |  | Conventional (29) | 0.33 ± 0.37 | 35.0 | 14.0 | 10.3 |  | 0 |  |
| Shahnazaryan et al, 2020 | 3 | 114 | 12 | Glass injector | DMEK (34) | 0.04 | 33 | 2.9 | 0 |  | 8.8 |  |
|  |  |  |  |  | Triple-DMEK (80) | 0.004 | 41 | 2.5 | 0 |  | 8.75 |  |
| Koechel et al,^92^ 2020 | 3 | 142 | 6 | Glass injector | Precut DMEK (44) | 0.4 | 42.5 | 32 | 5 |  |  | Raised IOP (9%); CME (7%) |
|  |  |  |  |  | Surgeon Prepared (98) | 0.3 | 36.3 | 35 | 1 |  |  | Raised IOP (6%); CME (2%) |
| Potts et al,^93^ 2020 | 3 | 400 | 6 | Glass injector | Preloaded DMEK (200) | 0.117 | 32.9 ± 18.5 | 12.5 |  |  |  |  |
|  |  |  |  |  | Preloaded DMEK (200) | 0.106 | 29.9 ± 16.4 | 17.5 |  |  |  |  |
| Böhm et al,^94^ 2021 | 3 | 68 | 6 | Glass injector | Preloaded DMEK (33) | 0.14 ± 0.13 |  | 13.2 | 2.63 |  |  |  |
|  |  |  |  |  | Preloaded DMEK (25) | 0.16 ± 0.15 |  | 33.3 | 0 |  |  | CME (3.3%) |
| Zwingelberg et al,^95^ 2021 | 3 | 402 | 12 | Glass injector | FECD (371) | 0.18 ± 0.27 | 41.2 | 29.8 |  |  |  |  |
|  |  |  |  |  | PBK (31) | 0.20 ± 0.14 | 43.5 | 27.5 |  |  |  |  |
| Jansen et al,^96^ 2021 | 3 | 125 | 24 | Glass injector | DMEK (125) | 0.18 ± 0.24; ≥20/20 (27.5%); ≥20/25 (55%); ≥20/30 (92.5%) |  | 11.2 |  |  |  |  |
| Fajardo-Sanchez,^97^ 2021 | 3 | 329 | 12 | Glass injector | Pseudophakic DMEK (218) |  |  | 21.1 | 7.8 | 6.9 | 3.2 |  |
|  |  |  |  |  | Triple-DMEK (111) |  |  | 17.1 | 4.5 | 2.7 | 1.0 |  |
| Guerra et al,^98^ 2011 | 4 | 136 | 12 | ICL injector and IOL injector | DMEK (136) | 0.07 ± 0.09; ≥ 20/20 (41%); ≥ 20/25 (80%); ≥20/30 (90%) | 36 ± 20 | 62.0 | 8.1 | 0.7 | 5.1 |  |
| Laaser et al,^99^ 2012 | 4 | 61 | 6 | IOL injector | DMEK (61) | 0.19 ± 0.22; ≥ 20/40 (81.4%); ≥20/25 (37.0%) | 39.8 | 73.8 |  |  |  |  |
| Parker et al,^100^ 2012 | 4 | 52 | 6 | Glass injector | Phakic DMEK (52) | ≥20/40 (100%); ≥20/25 (85%); ≥20/20 (67%) | 35.4 | 4.0^†^ | 0 | 0 | 0 |  |
| Anshu et al,^101^ 2012 | 4 | 141 | 13 | IOL injector | DMEK (141) |  |  |  |  |  | 0.7 |  |
| Gorovoy et al,^102^ 2014 | 4 | 75 | 3 | IOL injector | DMEK (75) | 0.10; ≥20/30 (85%); ≥20/20 (29%) | 19.0 | 2.7 | 2.7 | 0 |  |  |
| Monnereau et al,^53^ 2014 | 4 | 431 | 6 | IOL injector, glass injector, IV catheter | DMEK (431) | ≥20/40 (81.8%); ≥20/25 (43.8%); ≥20/20 (18.8%) | 47 ± 20 | 20.4  (34.6^†^) | 2.3 | 6.3 | 3.7 |  |
| Burkhart et al,^103^ 2014 | 4 | 49 | 12 | IOL injector | Phakic DMEK (49) | 20/20^*^; ≥20/25 (92%) | 25.0^*^ | 33.0 | 0 | 0 |  | Cataract progression (76%) |
| Maier et al,^104^ 2014 | 4 | 117 | 12 | Glass injector | DMEK (117) | 0.22 ± 0.22 | 28.3 | 44.4 | 0 | 0 | 0.9 | Raised IOP (12.1%) |
| Feng et al,^105^ 2014 | 4 | 673 | 60 | ICL injector | DMEK (673) |  | 39.0 | 30.0 |  |  |  |  |
| Deng et al,^106^ 2015 | 4 | 40 | 5.3 | IOL injector | DMEK (40) | 20/20; ≥20/20 (51.1%); ≥20/25 (76.9%); ≥20/40 (87.2%) | 30.5 (range 3.8 to 67.4) | 27.5 | 2.5 | 0 | 5 |  |
| Rodríguez-Calvo-de-Mora M et al,^107^ 2015 | 4 | 500 | 6 | Glass injector | DMEK (500) | ≥20/25 (75.0%); ≥20/20 (41.0%) | 37.0 | 3.0 | 0.2 | 0.2 | 0.2 | Regraft (2.2%) |
| Bhandari et al,^108^ 2015 | 4 | 30 | 12 | Silicon injector | DMEK (30) | 0.21 ± 0.12 | 24 | 10 | 0 | 0 | 0 |  |
| Schoenberg et al,^109^ 2015 | 4 | 108 | 11.9 | IOL injector | DMEK + CS (108) | 0.0^*^ (range -1.125 to 0.30; IQR 0.0 to 0.10) | 29^*^ | 16 | 0 | 0 |  |  |
| Gorovoy et al,^110^ 2015 | 4 | 125 | 12 | IOL injector | DMEK (125) |  | 19 ± 10 | 5.0 | 3 |  |  |  |
| Ham et al,^111^ 2016 | 4 | 250 | 48 | Glass injector | DMEK (250) | ≥20/40 (96%); ≥20/25 (83.0%); ≥20/20 (54.0%) | 52.6 | 4.4  (27.2^†^) | 1.6 | 2 | 2.4 | Repeat graft (15.2%) |
| Siggel et al,^112^ 2016 | 4 | 120 | 12 | IOL injector | DMEK (120) | 0.12 ± 0.12 ; ≥20/40 (96%); ≥20/25 (69.0%); ≥20/20 (40.0%) | 52.8 ± 13.7 | 75.8 |  | 1.8 | 1.7 | Raised IOP (7.2%) |
| van Dijk et al,^113^ 2016 | 4 | 67 | 24 | Glass injector | DMEK (67) | 0.07 ± 0.11 |  | 16.4^†^ |  |  |  |  |
| Schlögl et al,^114^ 2016 | 4 | 97 | 53 | IOL injector | DMEK (97) | 0.18 ± 0.16; ≥ 20/40 (97%); ≥20/25 (57%) | 44 |  | 2 | 2 | 1 |  |
| Rose-Nussbaumer et al,^43^ 2016 | 4 | 42 | 6 | IOL injector | DMEK (42) | 0.09 ± 0.12 | 43 | 19 | 11.9 |  | 0 | CME (4.7%); Glaucoma (2.4%) |
| Bhandari et al,^115^ 2016 | 4 | 40 | 6 | IOL injector | DMEK (40) | 0.30 ± 0.07 | 24 | 5 |  |  | 0 |  |
| Debellemaniere et al,^116^ 2017 | 4 | 109 | 6 | Glass injector | DMEK (109) | ≥20/40 (50.5%); ≥20/25 (30.3%); ≥20/20 (18.3%) | 38 ± 17 | 18 | 3.7 | 0 | 0.9 |  |
| Oellerich et al,^55^ 2017 | 4 | 2363 | 6 | Glass injector and IOL injector | DMEK (2363) | ≥20/40 (75.4%); ≥20/25 (45.4%); ≥20/20 (25.8%) | 40 ± 19 | 20.1 | 7.0 | 3.4 | 1.5 | CME (3.2%) |
| Peraza-Nieves et al,^117^ 2017 | 4 | 500 | 24 | Glass injector | DMEK (500) | ≥20/40 (98%); ≥20/25 (82.0%); ≥20/20 (52.0%) | 45 ± 18 | 15.8^†^ |  | 1.4 | 1.4 |  |
| Showail et al,^118^ 2018 | 4 | 250 | 6 | Glass injector or IOL injector | DMEK (250) | 0.3^*^ [IQR 0.2 - 0.5] | 26.1^*^ | 15.6 | 6 |  | 0.8 |  |
| Heinzelmann et al,^44^ 2018 | 4 | 1047 | 2 | IOL injector | DMEK (1047) |  |  | 24.0 |  |  |  |  |
| Basak et al,^119^ 2018 | 4 | 100 | 6 | IOL injector | DMEK (100) | ≥20/25 (57.6%) | 26.9 ± 13.40 | 4.0 | 1.0 |  |  |  |
| Kurji et al,^120^ 2018 | 4 | 28 | 12 | IOL injector | DMEK (28) | 0.09 ± 0.10 |  | 0 | 0 | 0 | 0 |  |
| Fajgenbaum et al,^121^ 2018 | 4 | 88 | 11 | Glass injector | DMEK (88) | ≥20/25 (86.0%); ≥20/20 (67.0%) | 35.0 ± 17 | 2.0 | 6 | 0 | 3 |  |
| Newman et al,^122^ 2018 | 4 | 111 | 6 | Glass injector | DMEK (111) |  | 30.9 | 14.4 | 0 |  |  |  |
| Price et al, ^123^ 2018 | 4 | 705 | 60 | IOL injector | DMEK (705) |  | 48 ± 19 |  | 6.2 | 1.3 | 2.6 |  |
| Schrittenlocher et al,^124^ 2018 | 4 | 1340 | 12 | IOL injector | DMEK (1340) | 0.10 ± 0.08 | 38 ± 15 | 43.4 | 0.36 |  | 1.34 |  |
| Droutsas et al,^125^ 2018 | 4 | 54 | 24 | Glass injector | DMEK (54) | 0.0^*^ (Range -0.20 - 0.70) | 44.6 |  |  |  |  |  |
| Godin et al,^126^ 2019 | 4 | 139 | 1 | Glass injector | DMEK (139) | 0.061; ≥20/25 (81.0%); ≥20/20 (61.0%) |  | 23.1 | 3.6 |  |  |  |
| Rickmann et al,^127^ 2019 | 4 | 254 | 13.2 | Glass injector | DMEK (254) |  | 29.6 | 21.7 | 1.6 | 1.6 |  |  |
| Sarnicola et al,^128^ 2019 | 4 | 78 | 12 | Glass injector | DMEK (78) | 0.0 ± 0.1 | 32.1 | 25.6 |  |  | 0 |  |
| Brockmann et al,^129^ 2019 | 4 | 108 | 12 | Glass injector | DMEK (108) | 0.11 ± 0.11 | 25.9 | 41.0 |  |  |  |  |
| Schaub et al,^130^ 2019 | 4 | 551 | 24 | IOL injector | DMEK (551) | 0.12 ± 0.10 |  |  |  |  |  |  |
| Livny et al,^131^ 2019 | 4 | 31 | 3 | Glass injector | DMEK (31) | 0.18 ± 0.14; ≥20/25 (44.4%) | 49 ± 20 | 16.0 | 3.2 |  |  |  |
| Basak et al,^132^ 2020 | 4 | 600 | 24 | IOL injector | DMEK (600) | 0.2 | 38.6 ± 14.3 | 3.8 | 0.5 | 1.0 | 1.2 |  |
| Siddharthan et al,^133^ 2020 | 4 | 230 | 24 | Plastic cartridge | DMEK (230) | 0.198 ± 0.23 | 47 ± 14 | 4.3 | 4.3 |  |  |  |
| Lekhanont et al,^134^ 2021 | 4 | 62 | 24 | Glass injector | DMEK (62) | 0.42 ± 0.58 | 45.9 | 12.9 | 16.1 | 1.6 | 4.8 |  |
| Marchand et al,^135^ 2021 | 4 | 85 | 12 | Glass injector | DMEK (85) | 0.18 ± 0.28 | 50 | 15 | 21 |  | 0 |  |
| Studeny et al,^136^ 2021 | 4 | 107 | 48 | Plastic cartridge | DMEK (107) | 0.19 ± 0.25 | 61.8 ± 20.0 | 57.9 | 14 | 2.8 | 1.9 | CME (1.9%) |
| **Endothelium In Techniques** | | | | | | | | | | | | |
| Ang et al,^33^ 2016 | 3 | 30 | 12 | Trifold Endoglide with D-Mat | DMEK (30) | ≥20/25  (66.7%)  ≥20/40  (89%) | 54.0 | 10  (16.7^†^) | 3 |  |  |  |
| Busin et al,^34^ 2018 | 3 | 46 | 6 | Trifold IOL injector | DMEK (46) | 20/25 | 29.5 ± 14.8 | 19.6 | 0 | 0 |  |  |
| Leon et al,^35^ 2018 | 3 | 173 | 0 | Trifold IOL injector with soft CL | DMEK (173) |  |  | 34.1 |  |  |  |  |
| Tan et al,^137^ 2020 | 3 | 69 | 6 | Trifold DMEK Endoglide | DMEK (69) |  | 33.6 | 11.6 | 1.5 |  |  |  |
| Yu et al,^138^ 2020 | 3 | 153 | 12 | Trifold IOL injector | DMEK (153) | ≥20/40 (100%); ≥20/25 (92%); ≥20/20 (66%); 0.018±0.069 | 29.6±14.3 | 27.4 | 0 | 6.5 | 0.7 |  |
| Woo et al,^139^ 2020 | 4 | 85 | 6 | Endoglide with donor stroma | DMEK (85) | ≥20/40 (95.7); ≥20/25 (44.7%) | 32.2±20.5 | 4.7 |  |  | 0 |  |
| Ighani et al,^140^ 2021 | 4 | 35 | 9 | Trifold DMEK Endoglide | DMEK (35) | 0.097^*^; ≥20/25 (52%) | 26.6 | 45.7 |  |  |  | Raised IOP (8.57%); CMO (2.86%) |
| Jabbour et al,^141^ 2021 | 4 | 33 | 6 | Trifold Spoon and infusion forceps | DMEK (33) | 0.09 ± 0.10 | 29.1 | 18.0 | 0 | 0 | 0 | Raised IOP (3.0%) |
| **Endothelium Out and Endothelium In Techniques** | | | | | | | | | | | | |
| Price et al,^36^ 2018 | 3 | 754 | 6 | IOL injector | Scroll endothelium out (Sugeon A) (245) |  | 28 ± 11 | 12 | 0.8 |  |  |  |
|  |  |  |  |  | Scroll endothelium out (Sugeon B) (161) |  | 30 ± 13 | 10 | 1.2 |  |  |  |
|  |  |  |  |  | Trifold endothelium in  (clear cornea) (172) |  | 28 ± 15 | 10 | 2.3 |  |  |  |
|  |  |  |  |  | Trifold endothelium in  (scleral tunnel) (176) |  | 27 ± 13 | 13 | 0.6 |  |  |  |

DMEK=Descemet membrane endothelial keratoplasty;CS=cataract surgery; BCVA=best corrected visual acuity; IOL=intraocular lens; PF=prednisolone acetate 1% drops; FML=fluorometholone 1% drops; LP=loteprednol etabonate 0.5% drops; ST=surgically treated glaucoma; MT=medically treated glaucoma; IV=intravenous; FECD=Fuchs’ endothelial corneal dystrophy; BK=bullous keratopathy; PBK= pseudophakic bullous keratopathy; CL= contact lens

^*^Median

^†^Graft detachment rate

^‡^Visual acuities reported as LogMAR or proportion of eyes ≥ specified Snellen acuity
